# RNA Interference Is Responsible for Reduction of Transgene Expression after Sleeping Beauty Transposase Mediated Somatic Integration

**DOI:** 10.1371/journal.pone.0035389

**Published:** 2012-05-03

**Authors:** Christina Rauschhuber, Anja Ehrhardt

**Affiliations:** 1 Max von Pettenkofer-Institute, Department of Virology, Ludwig-Maximilians-University Munich, Munich, Germany; 2 Institute of Virology and Microbiology, Center for Biomedical Education and Research, Department of Human Medicine, Faculty of Health, University Witten/Herdecke, Witten, Germany; National Institute on Aging, United States of America

## Abstract

**Background:**

Integrating non-viral vectors based on transposable elements are widely used for genetically engineering mammalian cells in functional genomics and therapeutic gene transfer. For the Sleeping Beauty (SB) transposase system it was demonstrated that convergent transcription driven by the SB transposase inverted repeats (IRs) in eukaryotic cells occurs after somatic integration. This could lead to formation of double-stranded RNAs potentially presenting targets for the RNA interference (RNAi) machinery and subsequently resulting into silencing of the transgene. Therefore, we aimed at investigating transgene expression upon transposition under RNA interference knockdown conditions.

**Principal Findings:**

To establish RNAi knockdown cell lines we took advantage of the P19 protein, which is derived from the tomato bushy stunt virus. P19 binds and inhibits 21 nucleotides long, small-interfering RNAs and was shown to sufficiently suppress RNAi. We found that transgene expression upon SB mediated transposition was enhanced, resulting into a 3.2-fold increased amount of colony forming units (CFU) after transposition. In contrast, if the transgene cassette is insulated from the influence of chromosomal position effects by the chicken-derived cHS4 insulating sequences or when applying the Forg Prince transposon system, that displays only negligible transcriptional activity, similar numbers of CFUs were obtained.

**Conclusion:**

In summary, we provide evidence for the first time that after somatic integration transposon derived transgene expression is regulated by the endogenous RNAi machinery. In the future this finding will help to further improve the molecular design of the SB transposase vector system.

## Introduction

Over the recent years various improved recombinases for somatic integration into the host genome were developed. Predominant integration systems currently being explored in mammalian cells are the transposable elements represented by the Sleeping Beauty (SB) transposase, the Frog Prince (FP) transposon, the piggyBac transposable element and the bacteriophage-derived integrase PhiC31 for targeted integration [1], [2], [3], [4], [5], [6].All systems are widely being studied in multiple applications including gene-therapeutic applications and functional genomics [7], [8], [9], [10].

The SB transposase system represents one of the most prominent non-viral gene therapy vectors because it can efficiently and stably integrate therapeutic DNA into mammalian genomes. SB transposase is a synthetic transposable element derived from fish [2]. The integration reaction is based on a cut-and-paste mechanism, which leads to genomic integration of the gene of interest, flanked by the SB inverted repeats (IRs) into a TA-dinucleotide within the genomic DNA. Multiple SB-based animal studies demonstrated efficacy in mice including stable correction of genetic disorders in clinically relevant animal models [11], [12], [13], [14].

There is accumulating evidence in invertebrates that DNA transposition is regulated by the endogenous RNA interference (RNAi) pathway [15], [16], [17], a mechanism responsible for transcriptional [18], [19], [20] and post-transcriptional gene silencing [21], [22], [23]. In these systems endogenously produced 21- to 25-nucleotide long non-coding micro RNAs (miRNAs) play a major role in downregulation of the transposase leading to inhibition of transposition in the respective cell [17], [24]. However, only limited information is available regarding the potential influence of miRNA pathway on DNA transposons in mammalian cells. A recent study showed that the LINE-1 retrotransposition is suppressed by endogenous small interfering RNAs [25]. Hereby, bidirectional dsRNA transcripts from the transposon represent targets for the RNAi machinery indicating that the RNAi pathway controls transposition in mammalian cells.

Herein, we hypothesized that transgene expression upon SB-mediated somatic integration may be regulated by the endogenous miRNA pathway. Recent data demonstrated that IRs display inward transcriptional activities in eukaryotic cells [26], [27] and this convergent transcription may lead to formation of double-stranded RNA (dsRNA) templates for the endogenous RNAi machinery which could lead to epigenetic silencing of transgene expression. This hypothesis is further supported by recent studies showing that epigenetic transgene silencing and therefore control of transgene expression can be mediated by convergent transcription and it is speculated that the mRNA specific micro RNAs and components of the RNA interference pathway play a role in this process [19], [28], [29].

Our experimental approach was based on generation of RNAi knockdown cell lines based on the RNAi inhibitor protein P19 derived from the tomato bushy stunt virus [30], [31], which inhibits 21 nt long small-interfering RNAs (siRNAs) [32], [33], [34]. To ascertain the impact of the RNAi pathway on transgene expression upon SB-mediated transposition, we quantified successful transposition events under selection pressure after transposition of a transposon containing a resistance gene as a direct indicator for transgene expression. We found up to 3.2-fold enhanced number of colony forming units in our RNAi knockdown cells supporting our hypothesis that RNAi reduces transgene expression in mammalian cells.

## Results

### Generation and Characterization of RNA Interference Knockdown Cells Based on the RNA Interference Inhibitor Protein P19

There is evidence that SB transposase derived IRs display convergent transcriptional activities in eukaryotic cells [26], [27]. This inward transcription may lead to formation of double-stranded RNA templates for the endogenous RNAi machinery. These findings motivated us to investigate whether transgene expression upon SB transposase-mediated somatic integration in mammalian cells is influenced by these miRNAs. To address this question our experimental approach was to generate RNAi knockdown mammalian cell lines, which can be used for evaluation of transposon-derived transgene expression levels after somatic integration.

Our hypothesis that transgene expression upon Sleeping Beauty mediated transposition is regulated by the RNA interference pathway is based on the formation of dsRNAs due to IR driven convergent transcription. In order to confirm the existence of dsRNAs originated from the transpsoson, we transfected a SB expressing plasmid (pCMV-SB) together with a plasmid harbouring the transposon (pTnori) and as control the SB transposase alone (**Figure S1 A**). Small RNAs were isolated 2 and 6 days post-infection and treated with RNase A and DNase to destroy of single-stranded RNAs and DNA, respectively. After the polyadenylation process, dsRNAs were reverse-transcribed and the cDNA was analyzed by PCR. PCR analysis revealed dsRNAs directed against the SV40 promoter and the neomycin reporter gene (**Figure S1 B**), whereas no dsRNA for the SV40 polyA signal or any larger dsRNAs covering the entire transposon (data not shown) could be detected. Thus, we were able to show that dsRNAs are generated from the transposon donor vector after SB mediated transposition, which have the potential to mediate either transcriptional or post-transcriptional gene silencing.

To establish RNAi knockdown cell lines we took advantage of the P19 protein, which is derived from the tomato bushy stunt virus [30], [31]. It binds and inhibits 21 nucleotide (nt) long, small interfering RNAs (siRNAs) and was shown to sufficiently suppress RNAi [33], [34], [35]. To check whether P19 is functional, we performed reporter assays in the human embryonic kidney cell line HEK293. In this assay the abrogated effect of an established small inhibitor RNA (siRNA) against a reporter directly correlates with P19 activity. We transfected HEK293 cells with a luciferase expressing plasmid along with either a P19 expressing plasmid (p19Topo or p19HA) or a mutated and inactive version of P19 (p19mHA). Twenty-four hours later we transfected either a previously published siRNA against firefly luciferase (GL3) or an irrelevant siRNA and 48 hours after the first transfection round we performed luciferase assays [32]. Plasmid constructs used for this study are shown in **Figure S2 A.** We found that P19 could restore the siRNA-mediated downregulation of firefly luciferase expression to up to 85% (**Figure S2 B,** bars 4 and 6), whereas the mutated form of P19 (p19mHA) could not (**Figure S2 B**). Furthermore our data revealed, that the HA-tagged P19 molecules were not as sufficient in blocking activity of siRNAs as an untagged version of P19 (**Figure S2 B**; compare bars 6 [tagged] and 4 [untagged]). Nevertheless, P19 was effective in suppressing siRNA-mediated silencing in HEK293 cells.

**Figure 1:**
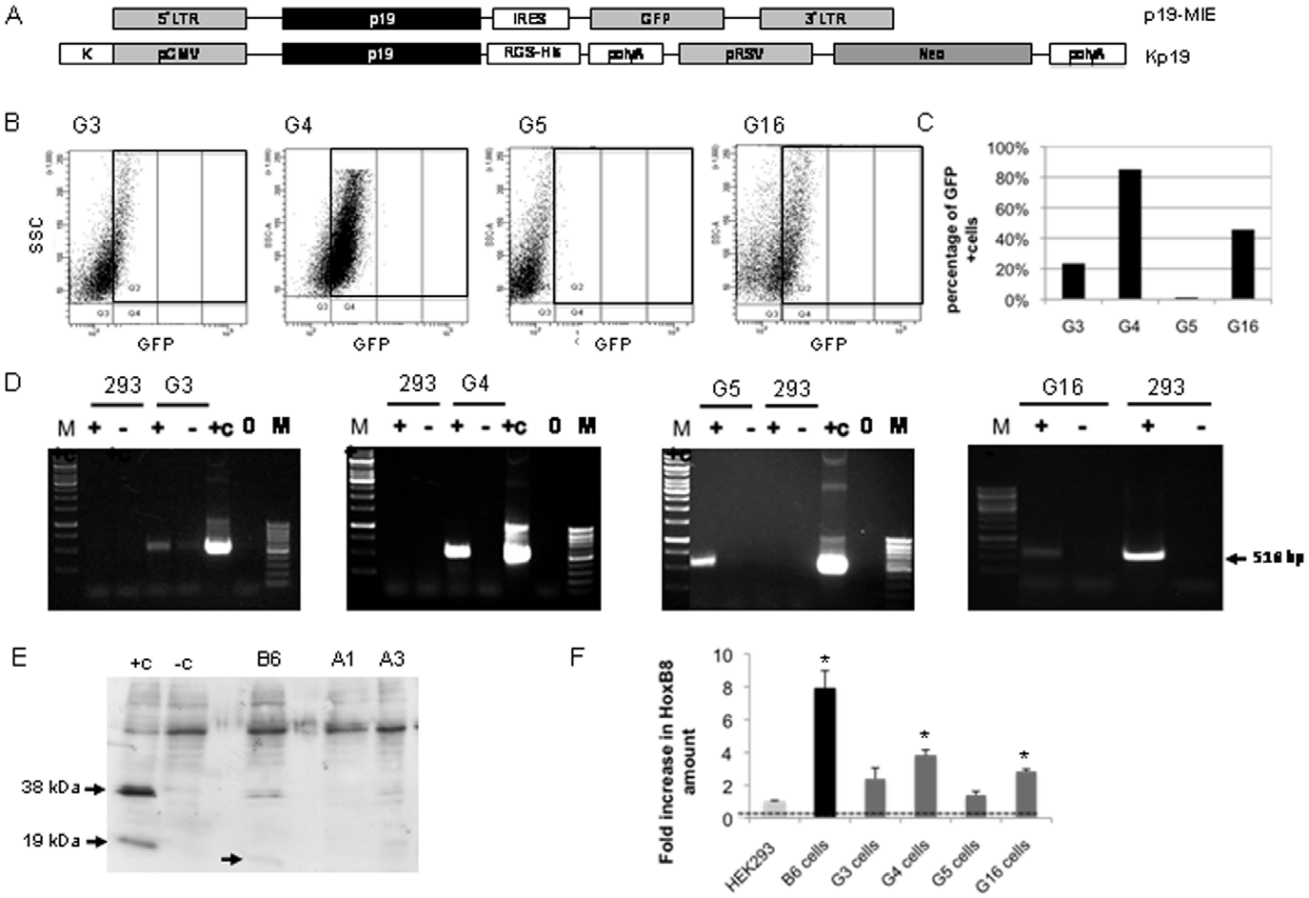
Generation and characterization of the RNAi knockdown cell lines. (A) DNA sequences used to generate stable *p19* expressing cell lines. Kp19 was used for stable plasmid transfection of HEK293 cells. The plasmid p19-MIE was used to produce a P19 expressing recombinant retrovirus for stable infection of HEK293 cells. K: Kozak sequence; pCMV: promoter of the cytomegalovirus; p19: p19 expression cassette; pRSV: promoter of the rous sarcoma virus; RGS-His: 6 histidin residues connected to the P19 protein by an arginin-glycin-serin motive; Neo: neomycin resistance cassette that mediates G418 resistance; poly A: polyadenylation signal derived from the simian virus; GFP: green fluorescent protein expression cassette; LTR: long terminal repeats; IRES: internal ribosome entry site. (B) Flow cytometric analysis of cell clones generated by retroviral transduction. Single cell clones from cell sorting were amplified and analysed by flow cytometry. Cells appearing in quadrant Q2 refer to GFP+cells. X-axis: GFP amount; Y-Axis: SSC: side scatter, to measure cell viability. **(**C) Quantitative analysis of GFP positive clones generated by cell sorting shown in **Fig. 1B**. (D) Expression of *p19* mRNA in the stable cell lines G3, G4, G5 and G16. The generated cDNA was used for PCR amplification with *p19* specific primers and a 519 bp band indicates positive cell clones. As positive control the p19 expression cassette from the plasmid Kp19 (+c) was amplified. +: sample with RT; −: sample without RT; 0: untreated HEK293 cells; M: marker. (E) Detection of P19 expression by Western Blot analysis in stable cell lines, which express the His-tagged version of the P19 protein. Monomeric and dimeric P19 molecules were detected using a peroxidase labeled anti-His antibody at 19 kDa and 38 kDa indicated by an arrow in the diagram. As positive control, HEK293 cells were transiently transfected with p19 expressing plasmids (left lane, +c) or mock transfected (-c). (F) Functionality of P19. RNA was isolated from HEK293, B6, G3, G4, G5, G16 cells and reverse transcribed. The cDNA was used for quantification of the HoxB8 mRNA amount by qRT-PCR. An increase in the HoxB8 level indicates a functional P19 protein because functional P19 inhibits miR169a- mediated downregulation of HoxB8. Normalization was performed by GAPDH measurement with GAPDH specific primers. The fold increase of the HoxB8 amount in the RNAi knockdown cell lines was determined in a semi-quantitative manner. *: p-value<0.05.

In order to analyze the effect of RNAi on SB transposition we established RNAi knockdown cell lines stably expressing P19. For this purpose we followed two strategies. In a first approach we infected HEK293 cells with a retrovirus expressing P19 as an un-tagged version and a green fluorescent protein (GFP) encoding cDNA (p19-MIE, **Figure 1A**). Using an un-tagged version of P19 could be advantageous because the tagged version of the P19 protein may be less biologically active compared to an un-tagged version (see also **Figure S2 B**). Upon retrovirus transduction, single cell clones expressing GFP were isolated by fluorescence-activated cell sorting and individual cell clones expressing GFP were amplified without selection. By this method we could establish 6 cell clones (G3, G4, G5, G6 G14 and G16) of which 4 clones (G3, G4, G5, G16) expressed GFP as analyzed by flow cytometry (**Figures 1B and 1C**). Clones G4 and G16 showed highest levels of GFP expression and RT-PCR analysis of cDNA generated from the GFP positiv cell lines could demonstrate expression of the untagged p19 mRNA (**Figure 1D** **)**. The second approach was based on stably transfecting the *p19* and *neomycin* resistance gene encoding plasmid Kp19 into HEK293 cells (**Figure 1A** **,** bottom panel). After plasmid transfection and subsequent G418 selection (500 µg/ml), 15 single, neomycin resistant HEK293-based cell clones were isolated and amplified. To analyze p19 expression, we performed Western Blot analysis using a peroxidase labeled anti-His antibody and found that cell clone B6 showed highest expression levels of both monomeric and dimeric P19 (**Figure 1E**) in comparison to two other cell clones (A1 and A2).

To investigate whether all generated cell lines express a functional P19 protein, we chose the HoxB8 gene as a marker. The *hoxB8* gene encodes a homeobox protein, a transcription factor that is only active during development. In differentiated cells HoxB8 is permanently suppressed by the endogenous miRNA miR-196a [36] (personal communication, Charles H. Lecellier, Institut de Génétique Humaine, Montpellier, France). Thus, if P19 is functional in our generated cell lines, HoxB8 expression should be upregulated, which can be measured by quantitative Real-Time PCR. By this approach we could show a 3.5 to 8-fold upregulation (p-value<0.05) of HoxB8 in the stable cell clones G4 and B6, respectively, clearly indicating that P19 is a sufficient inhibitor of the RNAi mechanism in this established cell lines (**Figure 1F**). However, all other clones showed only slight or no increase in HoxB8 amount (**Figure 1F**). Therefore, the following studies were performed using the RNAi knockdown cell lines B6 and G4.

### Influence of the RNA Interference Pathway on Transposon-derived Transgene Expression after Transposition

**Figure 2:**
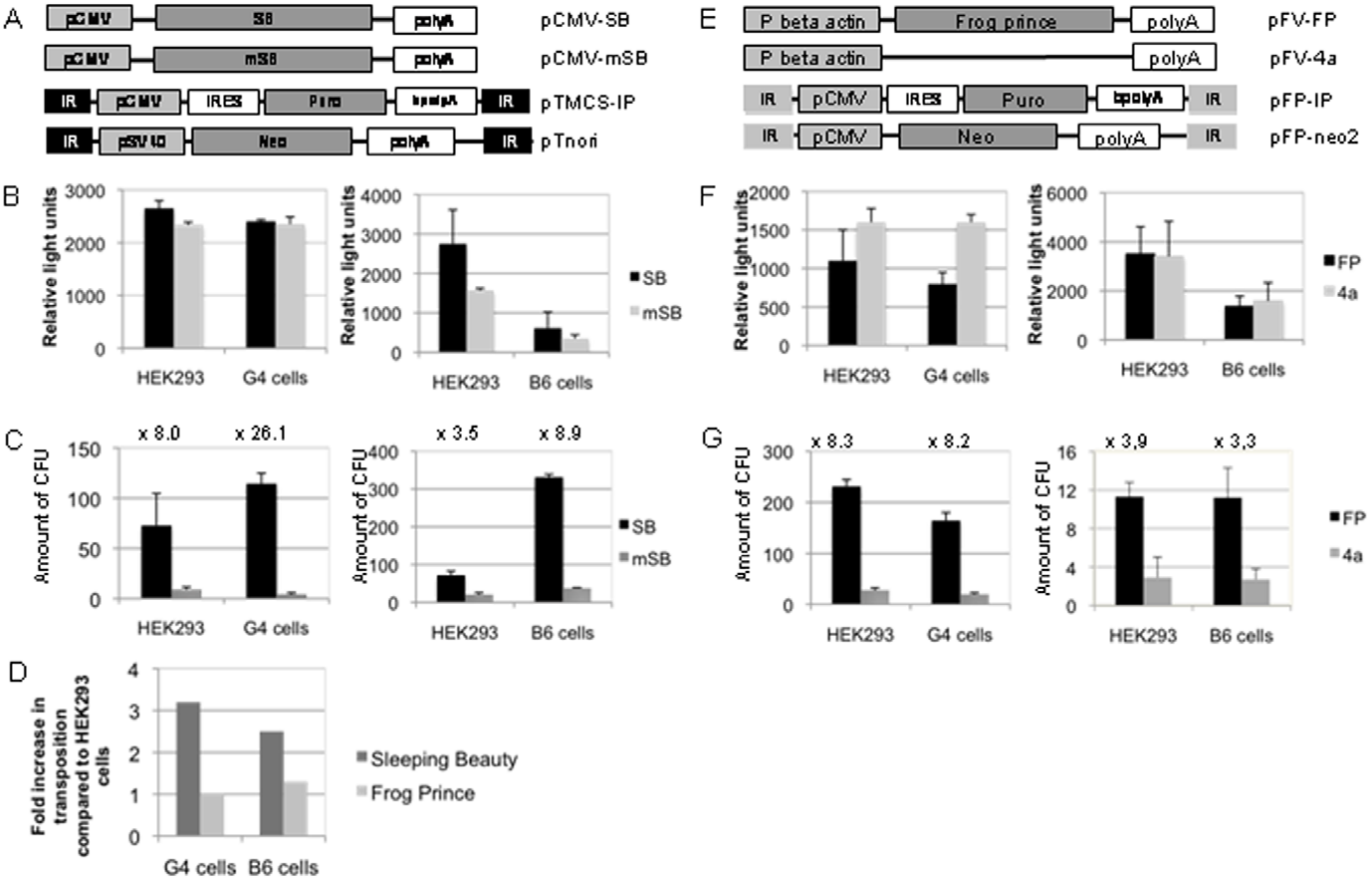
Influence of the RNA interference (RNAi) pathway on integration events mediated by Sleeping Beauty (SB) transposase and Frog Prince (FP) transposase. (A) Shown are all DNA constructs used to analyze the influence of the RNAi pathway on SB mediated transposition in HEK293 cells and the RNAi knockdown cell lines G4 and B6. The vector pTnori with the neomycin resistance gene was used as a transposon donor vector in G4 cells and pTMCS-IP was used in B6 cells. pCMV: major immediate early promoter/enhancer; IRES: internal ribosome entry side of the encephalomyocarditis virus (ECMV); Puro: puromycin-N-acetyl-transferase gene mediates puromycin resistance; bpolyA: bovine growth hormone A polyA signal; polyA: SV40 polyA signal; Neo: neomycin resistance cassette mediating G418 resistance; SB: SB transposase; mSB: mutated version of the Sleeping Beauty transposase; IR: inverted repeats recognized by SB. (B) Analysis of transfection efficiencies in HEK293, G4 (left panel) and B6 cells (right panel) two days post-transfection of the DNA constructs used for SB-mediated transposition. Instead of 100 ng stuffer plasmid as used for integration assays, 100 ng of the renilla luciferase expressing plasmid pRL-TK were transfected into 6-well plates and relative light units were determined. For details please refer to the material and methods section. The amount of relative light units correlates with the transfection efficiency. (C) Total number of colony forming units (CFUs) after SB transposase mediated integration in HEK293 cells and the RNAi knockdown cell lines G4 (left panel) or B6 (right panel). The fold increase in transposition events compared to the inactive protein is shown in numbers above the respective bars. All experiments were performed in triplicates and were statistically relevant (p-value<0.05). *: p-value<0.05. (D) The fold increase in integration events directly comparing the parental cell line HEK293 and the respective RNAi knockdown cell line for the SB transposase and the Frog Prince (FP) transposase are shown. (E) Shown are all DNA constructs used to analyze the influence of the RNAi pathway on FP mediated transposition in HEK293 cells and the RNAi knockdown cell lines G4 and B6. The transposon donor vector pFP-IP with the puromycin resistance gene was used in B6 cells and pFP-neo2 was used in G4 cells. P beta actin: beta actin promoter; FP: Frog Prince transposase; 4a: empty vector without FP transposase; IR: inverted repeats recognized by FP. (F) Analysis of transfection efficiencies in HEK293, G4 and B6 cells for the FP transposase system two days post-transfection. The amount of relative light units correlates with the transfection efficiency. (G) Total number of CFUs after Frog Prince mediated transposition in HEK293 cells and the RNAi knockdown cell lines G4 (left panel) or B6 (right panel). The fold increase in transposition events is expressed in numbers above the respective bars. The data comparing the active transposase with its inactive version within one cell line are statistically relevant (p-value<0.05). All experiments were performed in triplicates. * p-value<0.05.

As a next step we analyzed the influence of the RNAi pathway on transgene expression upon transposase mediated transposition in our RNAi knockdown cell lines. As described previously, we performed colony-forming assays (CFAs) [11], which allow for selection and quantification of transposition events in eukaryotic cells. We hypothesized that surviving cell clones expressed as colony forming units (CFU) after transposition correlate with transgene expression levels since no or low resistance gene expression levels result in cell death due to the selection pressure. Plasmids used for the analysis of SB transposition are shown in **Figure 2A**.

Since these CFAs are based on plasmid transfection into eukaryotic cells, we initially analyzed plasmid transfection efficiencies because the knockdown of the RNAi pathway potentially may have influenced the uptake of plasmid DNA. Therefore, we co-transfected a reporter plasmid expressing renilla luciferase and determined transfection efficiencies for each experiment performed in HEK293, G4 and B6 cells. We found that transfection efficiencies were comparable in the RNAi knockdown cell line G4 and the parental cell line HEK293 (**Figure 2B**, left panel). Although transfection efficiencies were different in the cell line B6 and the parental cell line, the ratios between the groups, which received the functional protein (SB) compared to the mutated version (mSB) were similar (**Figure 2B**, right panel). As described below we also considered this in our transposition analysis.

To analyze the efficiency of SB-mediated transposition, we co-transfected the RNAi knockdown cell lines G4 and B6 and the parental HEK293 cell line with the respective transposon donor vector (pTnori or pTMCS-IP) and either a plasmid encoding wild type SB (pCMV-SB) or the inactive version of the SB transposase (pCMV-mSB) at a molar ratio of 1∶4 (SB : transposon donor vector, **Figure 2A**). For CFAs performed in neomycin resistant B6 cells we utilized the transposon donor vector pTMCS-IP (**Figure 2A**) containing a puromycin resistance gene and for the G4 cell line we transfected the transposon donor plasmid pTnori (**Figure 2A**) encoding a neomycin resistance gene. After two weeks of G418 or puromycin selection the total number of colony-forming units (CFU) was quantified by counting colonies after methylene blue staining. All experiments were performed in triplicates and the average results of three independent experiments are shown.

To evaluate the results of the CFAs, we first analyzed the transposition rates in the RNAi-deficient cell lines and the parental cell line HEK293, independently. For each cell line and condition (SB or mSB) the amount of CFUs was quantified and transposition efficiencies were then expressed as the fold increase of SB-mediated integration events compared to cells, which received the inactive mSB. Importantly, this also takes into account variables like different cell states, cell growth or drug resistance. As displayed in **Figure 2C** this ratio of CFUs between functional (SB) and inactive (mSB) SB transposase was 8.0 and 3.5 in HEK293 cells, 26.1 in G4 cells and 8.9 in B6 cells, respectively. Afterwards, these data were used to directly compare colony-formation in the RNAi knockdown and HEK293 cells. In addition, the different transfection efficiencies were included in the calculation. As summarized in **Figure 2D** (dark grey bars) we found that in contrast to the parental cell line HEK293, SB-mediated integration showed at average an increase of 3.2-fold in the RNAi knockdown cell line G4 and an enhancement of 2.5-fold in the B6 cell line (p-value<0.05). Hence, we concluded that the RNAi pathway may interfere with transposon-derived transgene expression in mammalian cells and it could be speculated that epigenetic silencing, a process which as mentioned above can be regulated by the RNAi pathway, is responsible for modified transgene expression in P19 expressing cells.

Previous studies suggested that the Frog Prince (FP) transposon does not display convergent transcription, thus this system may not be influenced through the RNAi pathway [26]. Therefore, we speculated that the FP transposon system could be potentially used as a negative control for expression without impact of the RNAi pathway. FP is a reconstructed transposon from the Northern Leopard Frog *Rana pipiens* displaying high transpositional activity in vertebrate cells [4]. Similar to SB it works by a cut-and-paste mechanism leading to somatic integration into TA dinucleotides [4]. Plasmids used for the analysis of FP transposition in our RNAi knockdown cell lines are shown in **Figure 2E**. As for the SB transposase we first analyzed transfection efficiencies for all cell lines either receiving the active FP or an empty control vector (4a). Comparable to the results obtained for SB, the ratios for the transfection efficiencies were comparable for HEK293 cells and G4 cells (**Figure 2F**, left panel) and lower in the cell line B6 if directly compared to the parental cell line (**Figure 2F**, right panel).

In concordance to our studies utilizing the SB transposase system, we performed CFAs with the FP system in our RNAi knockdown cells G4 (**Figure 2G**, left panel) and B6 (**Figure 2G**, right panel) and the parental HEK293 cell line. The experimental setup was identical to the one used for SB and the total number of colonies for all cell lines are shown in **Figure 2G** **.** As displayed in **Figure 2G** the ratios of CFUs between functional (FP) transposase and the empty control vector (4a) were 8.3 and 3,9 in HEK293 cells, 8.2 in G4 cells (left panel) and 3,3 in B6 cells (right panel), respectively. Therefore, as hypothesized we could not detect a significant difference in CFU ratios in the RNAi knockdown cell lines in comparison to HEK293 cells after transfection of the FP expressing plasmid and the donor plasmids pFP-IP or pFP-neo2 at a molar ratio of 1∶5 (pFV-FP: substrate plasmid) (**Figure 2G**). The fold change in CFUs after FP transposition in G4 (similar) and B6 cells (up to 1.3-fold increase; p-value>0.05) in direct comparison to HEK293 cells is summarized in **Figure 2D** (bright grey bars). We concluded that transgene expression upon FP transposase mediated integration is not influenced by the RNAi machinery. Notably, the comparative analysis of the two experiments using Frog Prince and SB, in which we compared transposition ratios in the parental and the RNAi negative cell lines takes into account different conditions used, such as different promoters or in the case of FP using an empty control vector.

### The Effect of the RNAi Interence Pathway on Transposon-deirved Transgene Expression can be Abrogated by Insulator Sequences

**Figure 3:**
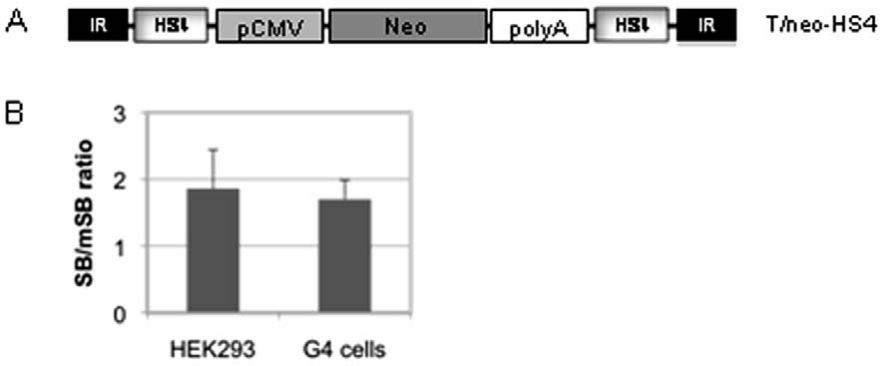
Analysis of the effect of insulator sequences on SB mediated transposition in HEK293 cells and the RNAi knockdown cell line G4. (A) DNA construct used for the study. pCMV: major immediate early promoter/enhancer; polyA: poly adenylation signal of the simian virus 40; SB: Sleeping Beauty (SB) transposase; mSB: non-functional version of the SB transposase; neo: neomycin re*sistance cassette; HS4: chicken insulator.* (B) The substrate plasmid T/neo-HS4 was transfected into HEK293 or G4 cells along with either the functional Sleeping Beauty transposase (SB) or the inactive version of SB (mSB). After 2 weeks of G418 selection, cells were stained and blue colonies were counted. The ratios of transposition events (SB to mSB) in G4 cells and HEK293 cells are displayed.

In order to further characterize the observed phenomenon in our RNAi knockdown cell lines on a molecular level, we performed CFAs and quantified SB-mediated transposition events from the substrate plasmid T/neo-HS4, containing two chicken β-globin insulating sequences (cHS4) [27]. There is evidence that these insulating sequences significantly reduce chromosomal position effects caused by enhancers present in the genome and it was shown that cHS4 sequences reduce epigenetic silencing based on hyperacetylation of histones H3 and H4 and reduction in CpG methylation [37], [38]. We performed CFAs transfecting T/neo-HS4 (**Figure 3A**) either with the functional SB or the inactive version of SB (mSB) in HEK293 cells and G4 cells using comparable conditions as in the experiments shown in **Figure 2**. After two weeks of G418 selection no significant differences in the ratio of transposition frequencies for the SB and the mSB group between HEK293 cells and the RNAi knockdown cell line G4 were observed (**Figure 3B**), indicating no RNAi-mediated decrease of transposon-derived transgene expression when using the cHS4 insulating sequences. This experiment supports the notion that really the RNAi mediated inhibition of the epigenetic mechanism, such as methylation of DNA or histones or deacetylation of DNA, drives downregulation of expression derived from integrated transposons.

### Transposon-derived Transgene Expression Levels are Higher in RNA Interference Knock-down Cells

**Figure 4:**
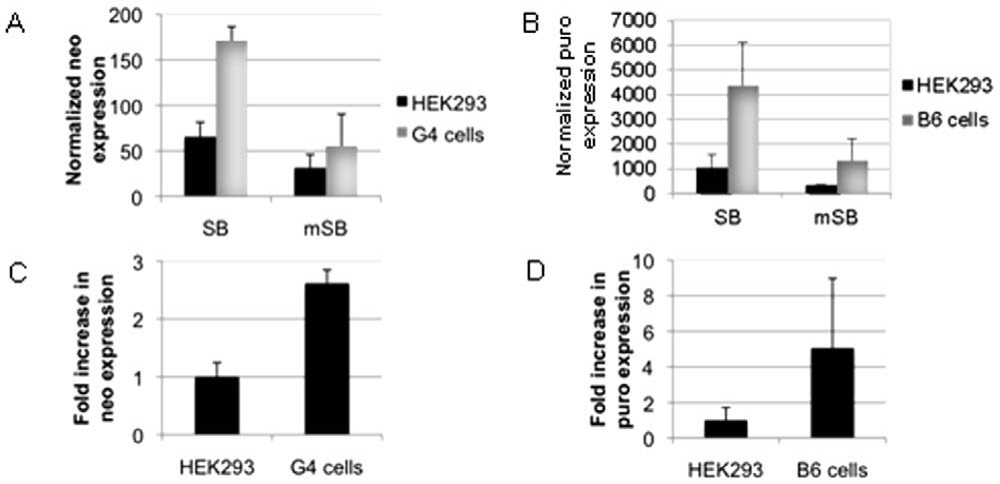
Quantification of transposon-derived transgene expression after Sleeping Beauty (SB) mediated transposition in HEK293 cells and the RNAi knockdown cell line G4 and B6. Transposon donor plasmids (pTnori for G4 cells and pTMCS-IP for B6 cells) and active or inactive SB transposase encoding plasmids (pCMC-SB and pCMV-mSB) were co-transfected into HEK293 cells and RNAi knockdown cell lines G4 and B6. Ten days (G4 cells) and 21 days (B6 cells) post-transfection, RNA was isolated from non-selected cells and reverse transcribed. The cDNA was then subjected to quantitative Real-Time PCR using neomycin or puromycin specific primers. Results were normalized to expression of 1000 human beta2 microglobulin RNA molecules. (A) Normalized neomycin (neo) expression in G4 cells compared to HEK293 cells. (B) Normalized puromycin (puro) expression in B6 and HEK293 cells. (C) The fold increase in neomycin expression is shown. Transgene expression in HEK293 cells was set to 1. (D) The fold increase in puromycin expression levels is is displayed. All data are statistically relevant (p-value<0.05).

In order to confirm our hypothesis that decreased transgene expression from the integrated transposon is the cause of reduced colony formation after transposition, we analyzed transgene expression levels after transposition from the donor vectors pTnori and pTMCS-IP. Therefore, we measured neomycin and puromycin expression levels by quantitative Real-Time PCR (qRT-PCR) 10 days (G4 cells) and 21 days (B6 cells) post-transfection using neomycin or puromycin specific primers. Results were normalized to expression of 1000 human beta2 microglobulin RNA molecules. As shown in **Figures 4A and 4B** expression levels were higher in both RNAi knockdown cell lines G4 (**Figure 4A**, neomycin) and B6 (**Figure 4B**, puromycin) when directly compared to the parental cell line HEK293. The fold increase in neomycin and puromycin expression is displayed in **Figures 4C** (neomycin) and **4D** (puromycin), indicating up to 3- to 5-fold increased transgene expression levels compared to HEK293 cells, respectively. These results suggested that higher transposon derived transgene expression levels can be one reason for increased CFU obtained from CFA in RNAi knockdown cells. To rule out if SB activities itself were modified in our RNAi knockdown cell lines and therefore responsible for differences in transposition events, we measured SB transposase activities by quantifying transposase mediated excision efficiencies from transposon donor plasmids by qRT-PCR. However, no differences in excision activities were detected (**Figure S3**).

## Discussion

In conclusion, our results indicate that transgene expression upon SB-mediated transposition in human cells is regulated through the RNAi pathway. There are several mechanisms possible for this phenomenon including suppression of mRNA expression at the gene level by introducing repressive chromatin modifications or at the mRNA level based on mRNA degradation or inhibition of translation. With respect to the latter point, one explanation could be that formed dsRNAs are exported out of the nucleus into the cytoplasm [39], [40] and that these RNAs may serve as substrates for Dicer, an endonuclease, which cleaves the long dsRNAs into 21–23 nt long siRNAs [41], [42]. One strand of these siRNAs, the so-called guide strand can then be incorporated into the RNA-induced silencing complex (RISC) and direct siRNA mediated silencing of the transgene after somatic integration [41], [43], [44]. Transcriptional silencing can also be mediated by siRNAs directed against promoter regions or transcriptional start-sites [45]. In agreement with this latter point, we detected dsRNAs originating from the SV-40 promoter region (**Figure S1 B**).

Our experiments using the cHS4 insulating sequences capable of inhibiting epigenetic silencing revealed no enhanced number of CFUs in RNAi knockdown cells (**Figure 3**). This result indicated that epigenetic silencing, such as methylation of DNA or histones or deacetylation of DNA, could drive downregulation of expression derived from integrated transposons. This is further supported by the data from Garrison and colleagues as they demonstrated increased transgene expression after Sleeping Beauty mediated transposition by applying methyltransferase inhibitors [46]. In conclusion, the results indicate that post-transposition silencing could be the primary cause of transgene silencing after SB mediated transposition. However, it remains to be analyzed whether and to which extent histone or/and DNA methylation occurs and this is also discussed quite controversial in the literature [18], [47]. One hypothesis is that first histone silencing occurs and this initiates the methylation of DNA sequences, which subsequently leads to heterochromatin formation. Chromosomal immunoprecipitation assays may solve the question in the case of the Sleeping Beauty transposition but other mechanisms such as de-acetylation cannot be excluded by this assay.

Notably, we not only detected dsRNAs against the SV-40 promoter region but also against the neomycin resistance gene and therefore, one could expect that the transgene itself is affected directly by post-transcriptional silencing mechanisms. Corresponding to knowledge about silencing mechanisms we speculate that at early time points post-transcriptional silencing based RNAi and dsRNAs plays an important role but persistent transgene downregulation may be mediated by epigenetic mechanisms resulting in heterochromatin formation.

Our hypothesis that IR driven dsRNA formation is responsible for increased numbers of CFUs in the RNAi knockdown cell line is also supported by other experiments performed in this study. Analysing the Frog Prince transposon system, for which no transcriptional activities derived from the IRs were demonstrated in the past [26], colony forming numbers after resistance gene selection were similar in the RNAi knockdown cell line and the parental cell line HEK293 (**Figure 2G**). Moreover quantification of transposon-derived reporter gene mRNA levels in G4 and B6 cells indicated significantly increased transgene expression levels in comparison to HEK293 cells (**Figure 4**). Thus, the obtained results confirm the assumption that transgene expression and not for instance increased transposase activities or levels contribute to the enhanced colony forming units detected in RNAi knockdown cell lines.

Interestingly, although the inhibition of the RNAi pathway seems to be influenced stronger in B6 cells compared to the G4 cells as indicated by the quantification of the HoxB8 expression levels (**Figure 1F**), we could not detect the same trend in the CFA analyzing transposition events. However, it is important to point out that different plasmid constructs were used to quantify integration events in the G4 and B6 cells. All substrate plasmids used for CFAs in B6 cells containing the puromycin expression cassette also harbour an intron as well as an IRES sequence. Since the transcriptional activity of the transposon IRs is relatively low [26], these components may function as insulators or they may interfere with the convergent transcription levels from the IRs after somatic integration. Additionally, transcriptional silencing may also explain the divergence we detected in between the assays in the G4 and the B6 cells. Different promoters and vector constitutions used in these assays may be affected in different dimensions by silencing processes. Nevertheless, the use of two independent plasmids may suggest that RNAi regulated mechanisms are conserved and independent of the transgene itself and the usage of longer and more complex transposons may reduce silencing processes.

In the present study we analyzed the commonly used two-component vector system for SB-mediated integration and observed an up to 3.2-fold increase CFUs in the RNAi knockdown cell line. However, effects may be increased when using the wild type SB transposase IRs with a 160 bp region between the left IR and the transposase translational start site with unknown function. This region was recently shown to enhance transcriptional activities from the left IR [27], which in turn may lead to an increased amount of dsRNA and therefore, potentially a more pronounced silencing effect.

In conclusion, our data indicate that the RNAi pathway regulates transposon-derived transgene expression potentially mediated by epigenetic silencing. In the future the established RNAi knockdown cell lines G4 and B6 can also be explored to study not only non-viral vectors and their regulation by the RNAi machinery, but also the influence of RNAi on viral vectors or viruses in general can be investigated. For instance herpesviruses and adenoviruses, which were shown to express siRNAs could be interesting candidates to address these questions [48], [49], [50], [51]. Last but not least our experimental approach based on expression of P19 may also be applied to study the interference of the RNAi pathway with viral or non-viral systems *in vivo* in a tissue specific manner. P19 could be expressed from a viral vector under the control of a tissue specific promoter, which may lead to tissue specific block of 21 nt long small interfering RNAs and therefore also inhibition of RNAi pathway.

## Materials and Methods

### Plasmid Construction

The plasmids pCMV-SB, pCMV-mSB, and pTnori were described elsewhere [11], [13]. The plasmids for Frog Prince transposition, pFV-FP, pFP-4a, pFP-neo, pFP-MCS and the T/neo-HS4 were kindly provided by Csaba Miskey and Zoltan Ivics (Max-Delbrück Center, Berlin, Germany)[4]. For construction of the plasmid pTMCS-IP, pIRESpuro2 (Clontech) was digested with *Xho*I, *Nru*I and *Nhe*I and the resulting 2678 bp fragment containing the puromycin resistance cassette was introduced into the *Bgl*II restriction site of pT-MCS [13] by blunt end cloning. Moreover, the same 2678 bp fragment was inserted into the *Eco*RI site of pFP-MCS resulting into the plasmid pFP-IP. For the plasmid pTMCS-RL, the plasmid pT-MCS was digested with *Xba*I and *Kpn*I, the ends were refilled using T4 DNA polymerase and subsequently the ends were religated again. The stuffer plasmid pBSP/PΔNot was described elsewhere [8], [12], [52]. Transfection efficiencies in RNAi knockdown cell lines were determined using the plasmid pRL-TK expressing renilla luciferase (Promega).

The plasmids used for the stable transfection of a P19 expression cassette were constructed with the gateway technology (Invitrogen). First, the p19 expression cassette was PCR amplified (without stop codon) with the primer Gp19forwK (5′AAAAAGCAGGCTCCGCCATGGAACGAGCTATACAAGGAA-3′) and the reverse primer Gp19rev (5′AGAAAGCTGGGTCGCTTTCTTTTTCGAAGGTTTGAG 3′) harboring a Kozak sequence for eukaryotic expression. To create homologous regions for recombination, attB specific primers were used for the nested PCR (One-for-all-forward: 5′-GGGGACAAGTTTGTACAAAAAAGCAGGCT-3′ and One-for-all-reverse: 5′-GGGGACCACTTTGTACAAGAAAGCTGGGTC-3′). Using BP clonase the PCR fragment was introduced into the attP sites of pDonr207 and in the second LR clonase reaction p19 was cloned into the destination vector pCR3.1 N-His. All Gateway compatible vectors were kindly provided by Armin Baiker (Max von Pettenkofer-Institute, Department of Virology, Ludwig-Maximilians-University Munich, Munich, Germany) [53], [54]. These cloning steps resulted into the plasmid Kp19, which was used to generate a stable p19 expressing cell line under G418 selection pressure (500 µg/ml) using Superfect transfection reagent (Qiagen). The p19 encoding retroviral vector p19-MIE, for generation of the stable cell line G4 by retroviral transduction, was produced by triple transfection of plasmids providing all necessary factors for retrovirus production, the gag-pol proteins as well as the VSV glycoprotein (all plasmids were kindly provided by Charles Lecellier (Institut de Génétique Humaine, Montpellier, France) and Olivier Voinnet (Institut de Biologie Moléculaire des Plantes, Strasbourg, France).

Plasmids for quantitative Real-Time PCR were constructed as follows. HoxB8 and GAPDH were amplified from human genomic DNA from HEK293 cells with the following primers: HoxB8forw_PD (5′-TGGAGCTGGAGAAGGAGTTC-3′), HoxB8rev_PD (5′-CTCCTCCTGCTCGCATTT-3′), hGAPDHforw (5′-TGCCTCCTGCACCACCAACT-3′) and hGAPDHrev (5′-CGCCTGCTTCACCACCTTC-3′) and either cloned into the *Eco*RI site of the the pBSP/PΔNot vector or into the commercial available pCR-Blunt II-TOPO (Invitrogen) vector, respectively, following the manufacturer instructions. Plasmid phB2m was a kind gift from Wenli Zhang (Max von Pettenkofer-Institute, Department of Virology, Ludwig-Maximilians-University Munich, Munich, Germany).

### Specific Analysis of dsRNAs Generated from the Transposon Donor Vector after SB Mediated Transposition

In order to confirm our fundamental hypothesis that dsRNAs are generated after SB mediated transposition we transfected 6-well plates of HEK293 cells at a confluency of 50% with 200 ng of the SB expressing plasmid pCMV-SB and 1000 ng of the transposon donor vector pTnori using FuGene 6 (Roche) following the manufacturer instruction. As a control HEK293 cells were used, which were only transfected with the SB expressing plasmid DNA. Either 2 or 6 days (control cells only 6 days) post-transfection small RNAs were isolated using the miRNeasy kit (Qiagen) also following the manufacturer instructions. To get rid of unwanted ssRNAs but protect dsRNAs, half of the eluted RNA was treated with 200 µg/ml RNase A and maintaining a 300 mM NaCl (DEPC-treated) concentration at 37°C. After 1 hour the mixture was purified using again the miRNeasy kit. Afterwards the remaining DNA was digested using DNaseA (Sigma-Aldrich) for 15 min at 37°C and either purified with the miRNeasy kit or using the provided stop solution to stop the reaction. Colomn purified RNAs were subjected to a polyadenylation reaction using the PolyA tailing kit (Ambion) and reverse transcribed by the *first strand cDNA synthesis kit* (NEB) with a polydTAdaptor primer(5′-GCGAGCACAGAATTAATACGACTCACTATAGG(T)12VN*-3′) [55]. RNAs treated with the stop solution were immediately used for reverse transcription but using the random primers provided by the NEB kit. As a control for possible DNA contamination one sample was not treated with the reverse transcriptase. The generated cDNA was then inserted into a PCR reaction using Taq polymerase with either the neomycin specific forward primer N1 (5′-CACCAGGGCAAGGGTCTG-3′) and the reverse primer N2 (5′-GCTCGTAGAAGGGGAGGTTG-3′) or the SV40 promoter specific primer SV40f (5′-GTTTAAACGCATCTCAATTAGTCAGC-3′) and SV40r (5′-GTTAATTAAAAGCTTTTTGCAAAAGCC-3).

### Generation and Characterization of A Stable p19 Expressing Cell Line by Retrovirus Transduction and Plasmid Transfection

For the production of the retrovirus harboring the *p19* cDNA, the plasmids p19-MIE, the gag-pol expressing plasmid and the VSV-glycoprotein expressing plasmid were triple-transfected into HEK293T cells using calcium phosphate transfection following the protocol from Promega (ProFection @ mammalian cells, Promega). Two days post transfection cells were harvested and filtered through a 0,45 µm filter (Millipore) to remove the remaining cellular material. The viral lysate was then supplemented with the same volume of Polybrene (Invitrogen), which facilitates cellular entry of the virus. Subsequently a 95% confluent 10 cm dish of 293T cells was transduced with the prepared retroviral lysate. Two days post infection, cells were sorted by fluorescent activated cell sorting (FACS) and single cell clones exhibiting GFP expression were sorted in a 96-well plate. These cell clones were amplified without selection. To check cells for GFP expression, several amplified cell clones were tested by flow cytometry using FACS-DIVA. In brief, stable GFP expressing cells as well as untreated HEK293 cells were harvested, washed once with PBS and centrifuged 3 min at 500 g. The cell pellet was then resuspended in PBS containing 0.1% FBS and supplied to the flow cytometer. Analysis was performed using FACS Diva software and the percentage of GFP positive cells within one population was calculated.

The second approach was based on stably transfecting the *p19* and *neomycin* resistance gene encoding plasmid Kp19 into HEK293 cells. HEK293 cells were cultured in DMEM supplemented with 10% FBS and 1% penecillin/streptomycin. Stable transfection of HEK293 cells with plasmid Kp19 at 70% confluency was performed using Superfect (Qiagen) according to the manufacturer instructions. Two days post transfection cells were seeded in 10 cm dishes at two concentrations (1×10^5^ and 1×10^4^ cells/dish) and 500 µg/ml G418 was added. After 14 days of selection, single cell clones were picked and amplified under G418 selection pressure.

### Western Blot Analysis to Investigate P19 Protein Expression

For Western Blot analysis a 6 cm tissue culture dish of the cell lines stably expressing a His-tagged version of P19, HEK293 control cells and transfected HEK293 cells with Kp19 were collected, washed once with PBS, treated with 300 µl NP-40 lysis buffer (150 mM NaCl, 1% NP-40, 50 mM Tris (pH 8.0)) and incubated for 30 min on ice. Protein lysates were separated on a 12% SDS-polyacrylamid gel and transferred to a PVDF membrane (Millipore). Detection of P19 was carried out by a peroxidase labeled anti-His antibody (Invitrogen) and ECL reaction (Amersham).

### P19 Specific Reverse Transcription PCR to Analyse Expression from Retrovirus Transduced Cells

The RNA of 3.2×10^6^ cells was isolated using the RNeasy kit (Qiagen) following the manufacturer instructions. After determining the RNA amount by measuring the optical density, 1 µg RNA was used for reverse transcription using polydT primer supplied in the *First strand DNA synthesis kit* (NEB). As a control, a reaction without the reverse transcriptase enzyme was performed to exclude non-integrated plasmids. The generated cDNA was then subjected to a PCR reaction with *p19* specific primers.

### Quantitative Real-Time PCR for Quantification of HoxB8, Neomycin and Puromycin Expression Levels, and Analysis of SB Transposition Efficiency

A quantitative Real-Time PCR (qRT-PCR) was performed using 4 µl of cDNA or 50 ng of genomic DNA, 0.5 millimolar specific primer (see plasmid construction section) and the *LightCycler FastStart* DNA Master^PLUS^ SYBR *Green* I (for HoxB8) (Roche) or with the *FastStart* Universal SYBR Green Master (Rox, Roche). The PCR reaction was run in the Light cycler 2.0 (Roche), or the TaqMan (Applied Biosystems), respectively. For semi-quantitative analysis, the vectors pHB2m, pFP-neo (neo qunatification), pIRESpuro (puro quantification) and pTMCS-RL (for transposition efficiency) were diluted and several 10-fold dilutions were used to create a standard curve. Quantification of neomycin, puromycin and SB transposition events were perfomed in a quantitative manner. Transgene expression levels for neomycin and puromycin were normalized to 1000 expressed hB2m molecules. For the neomycin PCR we used the forward primer N1 (5′-CACCAGGGCAAGGGTCTG-3′) and the reverse primer N2 (5′-GCTCGTAGAAGGGGAGGTTG-3′) resulting in a PCR product of 110 bp and to analyze puromycin expression levels we added primer P3 (5′-CTCGTCCTGCAGTTCATTCA-3′) and P4 (5′-AGACAATCGGCTGCTCTGAT-3′) to the PCR reaction amplifying a DNA fragment of 118 bp in length. For SB transposition efficiencies we measured the excision events from the transposon donor plasmid, which directly correlated with the amount of amplified fragments using the primer pair puc1 (5′-TACGCCAGCTGGCGAAAG-3′) and puc2 (5′?AGCTCACTCATTGGCAC-3′) [56]. For primer sequences used for quantification of HoxB8 expression please refer to the plasmid construction section.

### Colony Forming Assays (CFAs) for Quantification of Integration Events

For each colony forming assay (CFA) HEK293 cells and the RNAi knockdown cell lines B6 and G4 were seeded in 6-well plates. At 60% confluency cells were transfected with a total of 1 µg plasmid DNA and 10 µl transfection reagent (FuGene6, Roche). Experiments were performed in triplicates. Two days post-transfection cells of each 6-well were diluted (1∶30) into three 10 cm dishes. Selection with either 500 µg/ml G418 or 600 ng/ml puromycin was started 72 hours post-transfection and maintained for 2 weeks. The generated colonies were stained with methylene blue and counted.

The analysis of SB mediated transposition was performed by co-transfecting 50 ng pCMV-SB or 50 ng pCMV-mSB, 650 ng pBSP/PdeltaNot (stuffer DNA) and either 300 ng pTMCS-IP if B6 cells and puromycin selection were used or 300 ng pTnori or T/neo-HS4 if G4 cells and G418 selection were used. Frog Prince mediated transposition events were measured by transfection of 100 ng pFV-FP or 100 ng pFV-4a, 275 ng pBSP/PdeltaNot and either 625 ng pFP-IP in B6 cells or 625 ng pFPneo2 in G4 cells.

To evaluate transposition events, the ratio of colony-forming units between groups that received the functional transposase and groups that received a non-functional protein was determined. The fold increase in transposition events could be then identified by directly comparing this ratio in HEK293 cells and the RNAi knockdown cell lines.

For normalization of the results obtained after performing CFAs, transfection efficiencies were determined in the context of the CFA procedure by transfection of 100 ng pRL-TK plasmid instead of 100 ng of the stuffer plasmid pBSP/PΔNot. Two days post-transfection one sixth of the cell suspension was used to perform luciferase assays using the dual luciferase reporter assay (Promega). In order to include transfection efficienies in our calculation, we normalized the data received from the CFA to the results of the transfection efficiencies.

### Statistical Analysis

Statistical comparison was made by two-tailed Student T-test, and a value of p<0.05 was considered relevant compared with the respective control group.

## Supporting Information

Figure S1 **Detection of dsRNA derived from the transposon after SB mediated transposition.** (A) Shown are the two constructs used to analyse the existence of dsRNAs upon SB mediated transposition. pCMV: major immediate early promoter/enhancer; polyA: SV40 polyA signal; Neo: neomycin resistance cassette mediating G418 resistance; SB: SB transposase; IR: inverted repeats recognized by SB. (B) DsRNAs from the SB donor vector appear after SB mediated transposition. Two and six days after transfection of the SB encoding plasmid and the transposon encoding plasmid into HEK293 cells, cells were harvested and small RNAs were isolated. As control, cells, which were only transfected with the SB expressing plasmid were used. After Rnase A and DNase treatment, the RNA was reverse transcribed and subjected to PCR using primers specific for the SV40 promoter and the neomycin promoter (neo). DNA contamination was excluded by treating one sample without reverse transcriptase. M: Marker; d2: sample taken at day 2; d6 sample taken at day 6; c: control sample with only stuffer DNA transfected taken at day 6; -RT: sample taken at day 6 not supplemented with reverse transcriptase.
(TIF)

Figure S2 **Functionality of P19 in mammalian HEK293 cells.** (A) Plasmids used to analyze the functionality of P19 in mammalian HEK293 cells. pSV40: promoter of the simian virus-40; p19: p19 expression cassette; p19m: inactive *P*19 containing a*n* Arg72 to Glycin exchange; polyA: polyadenylation signal of the simian virus-40, HA: hemaglutinin-tag. (B) Luficerase assay to check the functionality of *P*19. Each sample analyzed contains the pGL3-Control plasmid (Promega). In addition, for samples displayed in black bars, either a non-specific stuffer plamsid, a functional p19 (p19Topo or p19HA) or the mutated version of p19 (p19mHA) was transfected together with a non-specific siRNA. In the samples referring to the grey bars, however, the GL3 specific siRNA was transfected together with the pGL3 alone or with one of the plasmids displayed in (A). RLU: relative light units; GL3: luciferase specific siRNA; *:p-value>0.5.
(TIF)

Figure S3 **Sleeping Beauty (SB) transposase excision activities are similar in normal and RNA interference knockdown cell lines.** To measure SB transposase activities in HEK293 cells and the RNAi knockdown cell lines G4 and B6, we determined transposase mediated excision efficiencies from transposon donor plasmids by quantitative Real-Time PCR (qRT-PCR) using the previously published primer pair puc1 and puc2 [56]. For generation of a standard curve the plasmid pTMCS-RL was used. Quantification was normalized to 1000 RNA molecules of human beta-2 microglobulin. (A) Quantification of SB transposase excision activities from the donor vector pTnori in HEK293 and G4 cells. The set-up of the qRT-PCR and the primer binding sites are schematically shown in the left panel. For the assay, the transposon donor vector pTnori was either co-transfected with the active SB transposase encoding plasmid (pCMV-SB) or the inactive transposase encoding plasmid (pCMV-mSB) into HEK293 and G4 cells. Two days post transfection whole genomic DNA was isolated and 50 ng genomic DNA was subjected to qRT-PCR, where excised and religated plasmids (referring to transposed plasmids, also demonstrated in the left panel) were quantified (right panel). (B) Excision activities from the donor vector pTMCS-IP in HEK293 and B6 cells. The experimental setup (left panel) was identical to the one described for the excision assay based on the transposon donor vector pTnori. The right panel shows measured excision activities.
(TIF)
