# Supplementary figures and images for: RNA Interference Is Responsible for Reduction of Transgene Expression after Sleeping Beauty Transposase Mediated Somatic Integration
Source: PLoS One. 2012 May 3;7(5):e35389. doi: 10.1371/journal.pone.0035389 (PMC3343047; doi:10.1371/journal.pone.0035389)

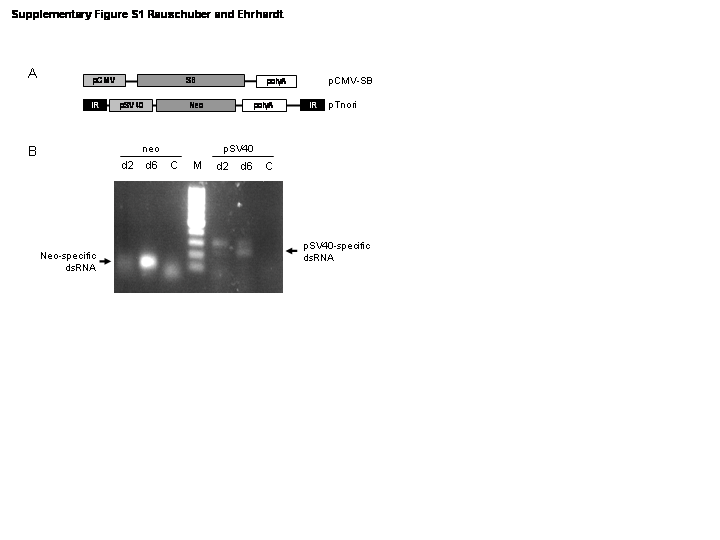

Supplement: Figure S1 — Detection of dsRNA derived from the transposon after SB mediated transposition. (A) Shown are the two constructs used to analyse the existence of dsRNAs upon SB mediated transposition. pCMV: major immediate early promoter/enhancer; polyA: SV40 polyA signal; Neo: neomycin resistance cassette mediating G418 resistance; SB: SB transposase; IR: inverted repeats recognized by SB. (B) DsRNAs from the SB donor vector appear after SB mediated transposition. Two and six days after transfection of the SB encoding plasmid and the transposon encoding plasmid into HEK293 cells, cells were harvested and small RNAs were isolated. As control, cells, which were only transfected with the SB expressing plasmid were used. After Rnase A and DNase treatment, the RNA was reverse transcribed and subjected to PCR using primers specific for the SV40 promoter and the neomycin promoter (neo). DNA contamination was excluded by treating one sample without reverse transcriptase. M: Marker; d2: sample taken at day 2; d6 sample taken at day 6; c: control sample with only stuffer DNA transfected taken at day 6; -RT: sample taken at day 6 not supplemented with reverse transcriptase. (TIF) [file pone.0035389.s001.tif]

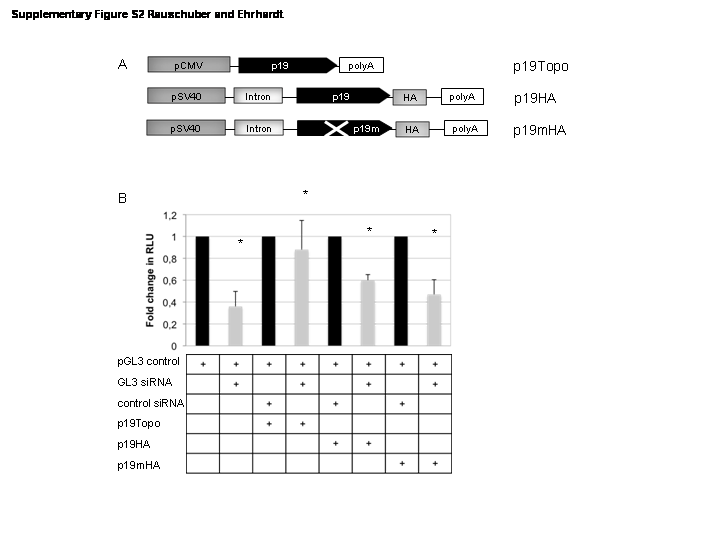

Supplement: Figure S2 — Functionality of P19 in mammalian HEK293 cells. (A) Plasmids used to analyze the functionality of P19 in mammalian HEK293 cells. pSV40: promoter of the simian virus-40; p19: p19 expression cassette; p19m: inactive P19 containing an Arg72 to Glycin exchange; polyA: polyadenylation signal of the simian virus-40, HA: hemaglutinin-tag. (B) Luficerase assay to check the functionality of P19. Each sample analyzed contains the pGL3-Control plasmid (Promega). In addition, for samples displayed in black bars, either a non-specific stuffer plamsid, a functional p19 (p19Topo or p19HA) or the mutated version of p19 (p19mHA) was transfected together with a non-specific siRNA. In the samples referring to the grey bars, however, the GL3 specific siRNA was transfected together with the pGL3 alone or with one of the plasmids displayed in (A). RLU: relative light units; GL3: luciferase specific siRNA; *:p-value>0.5. (TIF) [file pone.0035389.s002.tif]

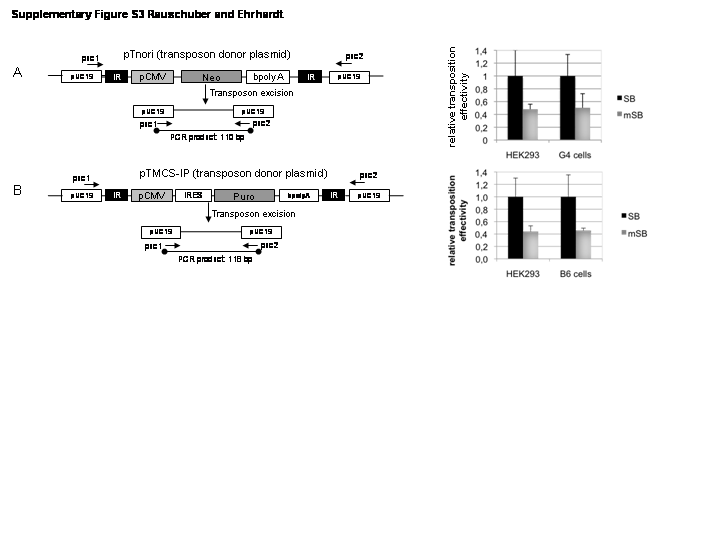

Supplement: Figure S3 — Sleeping Beauty (SB) transposase excision activities are similar in normal and RNA interference knockdown cell lines. To measure SB transposase activities in HEK293 cells and the RNAi knockdown cell lines G4 and B6, we determined transposase mediated excision efficiencies from transposon donor plasmids by quantitative Real-Time PCR (qRT-PCR) using the previously published primer pair puc1 and puc2 [56]. For generation of a standard curve the plasmid pTMCS-RL was used. Quantification was normalized to 1000 RNA molecules of human beta-2 microglobulin. (A) Quantification of SB transposase excision activities from the donor vector pTnori in HEK293 and G4 cells. The set-up of the qRT-PCR and the primer binding sites are schematically shown in the left panel. For the assay, the transposon donor vector pTnori was either co-transfected with the active SB transposase encoding plasmid (pCMV-SB) or the inactive transposase encoding plasmid (pCMV-mSB) into HEK293 and G4 cells. Two days post transfection whole genomic DNA was isolated and 50 ng genomic DNA was subjected to qRT-PCR, where excised and religated plasmids (referring to transposed plasmids, also demonstrated in the left panel) were quantified (right panel). (B) Excision activities from the donor vector pTMCS-IP in HEK293 and B6 cells. The experimental setup (left panel) was identical to the one described for the excision assay based on the transposon donor vector pTnori. The right panel shows measured excision activities. (TIF) [file pone.0035389.s003.tif]
